# A theory-based and data-driven approach to promoting physical activity through message-based interventions

**DOI:** 10.3389/fpsyg.2023.1200304

**Published:** 2023-07-27

**Authors:** Patrizia Catellani, Marco Biella, Valentina Carfora, Antonio Nardone, Luca Brischigiaro, Marina Rita Manera, Marco Piastra

**Affiliations:** ^1^Department of Psychology, Catholic University of the Sacred Heart, Milan, Italy; ^2^University of Pavia - Istituti Clinici Scientifici Maugeri IRCCS - Neurorehabilitation and Spinal Units, Pavia, Italy; ^3^Istituti Clinici Scientifici Maugeri IRCCS - Psychology Unit, Pavia, Italy; ^4^Department of Industrial, Computer and Biomedical Engineering, University of Pavia, Pavia, Italy

**Keywords:** physical activity, message intervention, framing, regulatory focus, artificial intelligence

## Abstract

**Objective:**

We investigated how physical activity can be effectively promoted with a message-based intervention, by combining the explanatory power of theory-based structural equation modeling with the predictive power of data-driven artificial intelligence.

**Methods:**

A sample of 564 participants took part in a two-week message intervention via a mobile app. We measured participants’ regulatory focus, attitude, perceived behavioral control, social norm, and intention to engage in physical activity. We then randomly assigned participants to four message conditions (gain, non-loss, non-gain, loss). After the intervention ended, we measured emotions triggered by the messages, involvement, deep processing, and any change in intention to engage in physical activity.

**Results:**

Data analysis confirmed the soundness of our theory-based structural equation model (SEM) and how the emotions triggered by the messages mediated the influence of regulatory focus on involvement, deep processing of the messages, and intention. We then developed a Dynamic Bayesian Network (DBN) that incorporated the SEM model and the message frame intervention as a structural backbone to obtain the best combination of in-sample explanatory power and out-of-sample predictive power. Using a Deep Reinforcement Learning (DRL) approach, we then developed an automated, fast-profiling strategy to quickly select the best message strategy, based on the characteristics of each potential respondent. Finally, the fast-profiling method was integrated into an AI-based chatbot.

**Conclusion:**

Combining the explanatory power of theory-driven structural equation modeling with the predictive power of data-driven artificial intelligence is a promising strategy to effectively promote physical activity with message-based interventions.

## Introduction

Regular physical activity is one of the cornerstones of a healthy lifestyle, as it has numerous benefits for health, well-being and reduced welfare costs (Ding et al., 2016; Marquez et al., 2020; WHO, 2020). To date, psychological research has addressed the issue of physical activity engagement using either a theory-based *or* a data-based approach (Yarkoni and Westfall, 2017; Dijkhuis et al., 2018; Aldenaini et al., 2020; Zhang et al., 2020), while only a small body of research fully combines the two approaches (Catellani et al., 2022). The present research aimed to combine the best of both worlds, combining the great explanatory power of a theory-driven approach with the great predictive power of a data-driven method. The former was achieved by testing the goodness of fit of a structural equation model based on established psychosocial theories. The latter was achieved by first transforming the structural model into a predictive Dynamic Bayesian Network (Dagum et al., 1995; Murphy, 2012) and then using the network through Deep Reinforcement Learning (Li, 2017; François-Lavet et al., 2018) to obtain a fast profiling method. This method enabled the selection of the most promising message wording for specific recipients and was later integrated into a chatbot to promote physical activity. Taken together, the theory-based and data-driven approaches shed new light on *what* causal relationships can be predicted in the field of physical activity promotion and *why* these specific causal relationships can be predicted. The fusion of these two approaches is relevant to both quantitative psychology (Yarkoni and Westfall, 2017) and the application of artificial intelligence techniques to the topic at hand (Adadi and Berrada, 2018).

Our study was part of the larger project RE-Hub-ILITY (Rehabilitative Personalized Home System and Virtual Coaching for Chronic Treatment in Elderly), funded by the Region of Lombardy (Italy) and carried out by a clinical institute led by a consortium of universities and private companies. The entire project involved both patients and the general population and aimed to develop feasible ways to increase the frequency of physical activity and evaluate their effectiveness using advanced technology. The outcome of the project was an integrated platform with wearable devices, range cameras, exergames, smartphone apps with virtual coaching systems, all connected via the cloud with control and monitoring functions. As part of this large and interdisciplinary project, the study presented in this paper aimed to develop an integrated approach to increase participants’ willingness to engage in physical activity by combining proven psychological theories with artificial intelligence tools.

## Theoretical background

### Elaboration likelihood model and message-induced emotions

According to the well-established Elaboration Likelihood Model (Petty and Cacioppo, 1986; Petty and Briñol, 2011), persuasion and message elaboration can follow a central or a peripheral route. On the former route, recipients engage in conscious and cognitively demanding processing, while on the latter they engage in superficial and resource-conserving processing. When a message is processed on the central route, the resulting persuasion process is likely to be longer lasting. Activation of the central route is stimulated by the receiver’s commitment to the message. A highly involved receiver is more likely to rely on the central route, where deeper processing triggers efficient and long-lasting attitude change.

Consistent with the Elaboration Likelihood Model, in the present study we expected that higher involvement of the recipient of the message would lead to deeper processing of the content of the message, which in turn would lead to greater intention to engage in physical activity, even after controlling for the level of prior intentions.

The persuasiveness of a message is also linked to the activation of negative or positive emotions. If a message is perceived as too aversive or too reassuring, it may trigger a defensive reaction in recipients or lead them not to comply with the message’s recommendation (Hovland et al., 1953; Leventhal, 1970). The reason for this is that emotions mediate the relationship between activation by the message and successful persuasion. For example, messages that elicit anger are likely to lead to reactance and, consequently, persuasion failure (Dillard and Shen, 2005; Rains and Turner, 2007; Carfora et al., 2021). Regarding positive emotions, several studies have highlighted that they are crucial to promote physical activity engagement (e.g., Dillard and Nabi, 2006; Conner et al., 2015; Kühne and Schemer, 2015).

In the present study, we expected that messages eliciting higher levels of composure would be less effective in getting participants to comply with the message’s recommendation, as they would not elicit the recipient’s commitment (Hovland et al., 1953; Leventhal, 1970). Instead, we expected that messages that inspired more hope would be more effective and would trigger the recipient’s commitment. Hope is not only beneficial in itself (e.g., Richman et al., 2005), but is also positively related to willingness to exercise regularly in university students (Moylan et al., 2022) and to increases in physical activity in children participating in a weight management program (Van Allen and Steele, 2012).

### Self-regulatory model of message framing

The persuasiveness of message-based interventions depends on how the specific messages are framed (e.g., Williams et al., 2019). Depending on whether the message is framed in terms of avoiding or obtaining the expected pleasure or pain, Cesario et al. (2013) classified messages into four categories. A *gain message* highlights the achievement of a positive consequence (e.g., “If you exercise regularly, you will enjoy the benefits of good health”). A *non-loss message* emphasizes the avoidance of an undesirable negative consequence (e.g., “If you exercise every day, you will avoid the risk of increased blood pressure”). A *non-gain message* emphasizes the possibility of foregoing a desirable positive consequence (e.g., “If you sit for many hours, you will miss out on the benefits of a healthy circulatory system”). Finally, a *loss message* emphasizes the triggering of a negative consequence (e.g., “If you sit for a long time every day, your lung capacity will decrease”).

This fine-grained taxonomy of persuasive messages has been suggested as a possible explanation for the mixed results of previous research on the effectiveness of message-based health promotion interventions (Gallagher and Updegraff, 2012; Rothman et al., 2020). Interestingly, evidence suggests that no specific frame is superior to others in this area and that the fit between the message frame and the given characteristics of the message recipient is the best candidate to explain effective persuasion (Yi and Baumgartner, 2009; Bertolotti et al., 2020).

### Regulatory focus theory

A key receiver trait known to affect responses to persuasive messages is the individual predisposition to support one of two distinct regulatory foci (Higgins, 1997; Cesario et al., 2013). People with a predominant *promotion focus* achieved self-regulation by actively seeking gains and achieving ideal desirable states. People with a predominant *prevention focus* achieved self-regulation by avoiding losses and striving to fulfill obligations and tasks. It can be assumed that the regulatory focus of the recipient of a message interacts with the frame of the message and enhances persuasion when the two fit together. Moreover, the effect that messages with different frames can have on people with a different regulatory focus is most likely mediated by the emotions triggered by contact with the message. From previous research (Brockner and Higgins, 2001), we know that promotion-focused people experience emotions that are on the continuum between happy and depressed (e.g., hope), while prevention-focused people experience emotions that are on the continuum between calm and excited (e.g., fear).

Building on the above, in the present study we expected that participants would respond emotionally to persuasive messages according to the continuum associated with their dominant focus. We expected calmness and hope to act as mediating factors between participants’ disposition in relation to regulatory focus and their engagement with the messages. Consistent with the regulatory focus literature presented above, we expected calmness to mediate the effects of prevention focus on message engagement and hope to mediate the effects of promotion focus on message engagement.

### The present study

With our study, we aimed to find out how physical activity can be effectively promoted using messages by combining the *explanatory power* of theory-driven structural equation modeling with the *predictive power* of data-driven artificial intelligence.

We first tested a structural equation model based on the theoretical framework described above. The model was designed to illustrate how exposure to messages can influence intention to engage in physical activity, triggering emotions that in turn influence engagement and message processing. The model had the two regulatory foci, i.e., promotion and prevention, as exogenous variables. It was expected that these two individual differences would predict the emotions experienced in response to being addressed by persuasive messages. We expected that emotions would mediate the effect of regulatory focus on perceived engagement with the messages. We also expected that higher involvement would lead to deeper processing of the message, which in turn would trigger higher intention to engage in physical activity. To ensure that regulatory focus, emotion, involvement and deep processing explained post-intervention intention without relying on high levels of pre-intervention intention, we introduced this variable as another predictor of final intention. In this way, the effect of deep processing could be interpreted as an effect of the intervention over and above the individual tendency of our participants before the intervention.

In addition to assessing the goodness of the model as a whole, we tested a series of hypotheses regarding the effect of calm and hope on message involvement as well as their mediating role in the relationship between regulatory focus and message involvement.

*H1*: Calm triggered by exposure to the messages reduces message involvement.

*H2*: Hope triggered by exposure to the messages increases message involvement.

*H3*: A prevention focus leads to lower calm and consequently higher message involvement.

*H4*: A promotion focus leads to higher calm and consequently lower message involvement.

*H5*: A prevention focus leads to lower hope and consequently lower message involvement.

*H6*: A promotion focus leads to higher hope and consequently higher message involvement.

A further goal of our study was to combine the *explanatory power* of theory-driven structural equation modeling with the *predictive power* of data-driven artificial intelligence. From this point of view, the SEM model would have formed the backbone of a more comprehensive predictive model, a Dynamic Bayesian Network (DBN). This model would have included additional variables that could be relevant to understanding which messages might best promote physical activity in people. Thus, while we used the SEM model to identify the effects of regulatory focus and emotions generally triggered by messages promoting physical activity, in the broader model used for the DBN we had the opportunity to include other dimensions related to participants and the different types of messages sent.

In terms of participant characteristics, the DBN integrated the variables of the SEM model with additional variables that previous research has shown to play a role in predicting engagement in physical activity, namely the psychosocial variables envisaged in the Theory of Planned Behavior (i.e., attitude, perceived behavioral control, and social norm; Ajzen, 1991; Conner and Armitage, 1998; Wolstenholme et al., 2021; Carfora et al., 2022) and socio-demographic variables (i.e., age, education, and gender). Such a model would therefore provide an answer to the following research question.

**RQ1**. How do socio-demographic variables, regulatory focus and Theory of Planned Behavior variables predict intention to engage in physical activity?

In relation to the messages promoting physical activity, the framing of the messages was included in the Dynamic Bayesian Network, i.e., the fact that the messages inserted in the message intervention were framed with a gain, non-loss, non-gain, or loss frame. The aim was to predict whether exposure to differently framed messages would differentially predict recipients’ responses, in terms of emotions, involvement, deep processing and intention change. We therefore formulated the following research question.

**RQ2**. How does exposure to differentially framed messages (i.e., gain, non-loss, non-gain, loss messages) predict emotions elicited by the messages, message involvement, deep processing, and intention to engage in physical activity?

A final goal of our study was to use the identified DBN via Deep Reinforcement Learning to develop an automated, rapid profiling procedure to determine the best messaging strategy based on the characteristics of each potential respondent. Such a procedure would enable us to answer our third research question.

**RQ3**. What questions, and in what order, need to be asked to quickly profile recipients and then send them messages designed to maximize the likelihood of increasing intention to engage in physical activity?

If the planned pathway to answering the three research questions described above proved adequate, we could claim to have found an effective way to harness the predictive power of data-driven artificial intelligence to effectively promote physical activity with message-based interventions.

## Methods

### Participants and procedure

This study was ethically approved by the Catholic University of the Sacred Heart (Milan) and the Scientific Committee of the Istituti Clinici Scientifici Maugeri (Pavia). The sample size required to test the theory-based structural equation model was estimated based on an *a priori* power analysis calculated using the *A-priori* Sample Size Calculator for Structural Equation Models by Soper (2021). The model involved the estimation of 11 latent variables based on 41 observed indicators (*α* = 0.05; *1-β* = 0.90; effect size = 0.30). The resulting required sample size was *N* = 238. To ensure the sample size despite any dropouts during the intervention phase, we included in the study more participants than required.

In February 2022, participants (all Italian individuals) were invited to participate in a university study through Prolific,^1^ a platform for online recreuitment designed for research. The research was advertised as research on lifestyle, about a total of 40 min in length, and distributed in 15 days. To be eligible for the study, participants had to be residents in Italy and have a Prolific record of at least 75% satisfactorily completed experiments.

All participants were informed that the study consisted of completing two questionnaires at different time points and receiving a message every day for a fortnight. Participants were randomly assigned to one of the four experimental conditions of the study (gain, non-loss, non-gain, and loss messages). Each participant was given an anonymous alphanumerical code to enter into the PsyMe app. PsyMe is a free smartphone app from the Catholic University of the Sacred Heart in Milan and the University of Pavia. It was developed to support scientific research in the field of social psychology and artificial intelligence. The PsyMe app respects the privacy and anonymity of participants thanks to the assignment of an anonymous code for each participant. It allows sending questionnaires, messages and push notifications to remind people to read the messages.

**Figure 1:**
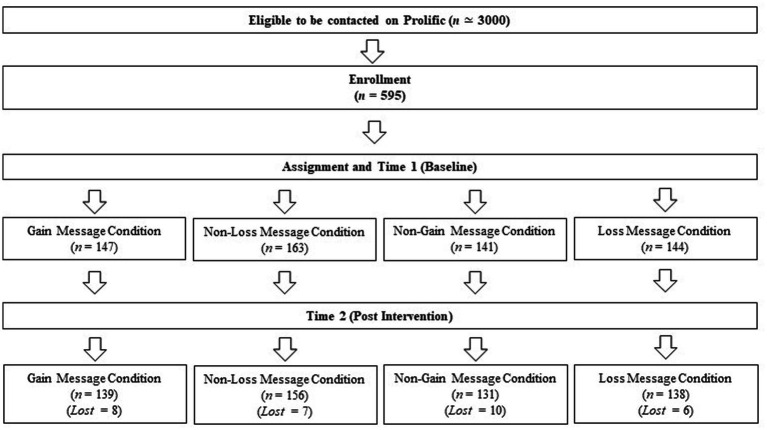
Participant flow chart.

Figure 1 shows the flow of participants during the study. The initial sample consisted of N = 595 healthy respondents recruited on Prolific. After excluding participants who dropped out of the study and did not provide information on post-intervention measures (*N* = 31), the final sample was *N* = 564. The sample was balanced in terms of gender (284 females, 280 males) and a wide age range was represented as participants ranged from 18 to 65 years old (*M* = 29.99, *SD* = 9.44).

Besides the Prolific sample of healthy participants, a further sample of patients diagnosed with cardiovascular problems (*N* = 41, randomly distributed across message conditions; 27 males, 14 females, ranging from 22 to 81 years old, *M* = 59.85, *SD* = 15.06) was employed in the study, to validate the elicited Dynamic Bayesian Network by an in/out analysis (see the related section of the Results). This sample of patients was recruited via Istituti Clinici Scientifici Maugeri.

### Pre-intervention measures

*Regulatory focus* was assessed by measuring participants’ tendency to avoid undesirable states (i.e., prevention; *α* = 0.83) and to achieve desirable states (i.e., promotion; *α* = 0.82) (adapted from Ferrer et al., 2017). *Cognitive* and *affective attitudes* were measured using a semantic differential scale (e.g., Caso et al., 2021; cognitive attitude: *α* = 0.87; affective attitude: *α* = 0.87). *Subjective norm* was assessed with three items (e.g., adapted from Carfora et al., 2020; *α* = 0.83). *Perceived behavioral control* was measured using five items (*α* = 0.90). *Intention* to engage in physical activity was assessed using three items (Catellani et al., 2021; *α* = 0.97). All of the above measurements were on a 7-point Likert scale. The full list of items can be found in Supplementary Table S1. Data collected included age, gender, education, and marital status.

### Message intervention

During the 2-week intervention, participants received a total of 14 persuasive messages daily via the PsyMe app. Participants were randomly assigned to one of four different message frames. The *gain messages* described the positive consequences of physical activity (e.g., “If you exercise regularly, you will improve your fitness”). The *non-loss messages* focused on avoiding undesirable consequences of not being physically active (e.g., “If you exercise regularly, you will avoid a decline in your fitness”). *Non-gain messages* focused on missing out on the positive consequences of physical activity (e.g., “If you exercise regularly, you will avoid the risk of cardiovascular problems”). Finally, the *loss messages* described the negative consequences of not exercising (e.g., “If you do not exercise regularly, you risk cardiovascular problems”). Each message focused on a physical, social, or mental benefit of regular exercise and was framed in a pre-factual (i.e., “if… then”) structure (Carfora and Catellani, 2021). The full list of messages can be found in Supplementary Table S2.

### Post-intervention measures

Each emotional response to the messages (*anger, anxiety, fear, hope*, and *calm*) was assessed using three items (adapted from Nabi et al., 2018; Seo and Dillard, 2019; anger, *α* = 0.91; fear, *α* = 0.82; anxiety, *α* = 0.80; calm, *α* = 0.86; hope, *α* = 0.86). *Involvement* was assessed using three items (Carfora and Catellani, 2021; e.g., *α* = 0.88). *Deep Processing* was assessed using five items (*α* = 0.88). *Intention* to engage in physical activity was assessed using the same three items used before the intervention (*α* = 0.96). All measurements were on a 7-point Likert scale. The full list of items can be found in Supplementary Table S1.

### Data analysis

After preliminary analyses, we first tested the quality of our explanatory theoretical model using structural equation modeling (SEM). The goodness of fit of our model and of two alternative models was tested using Chi-square and incremental goodness-of-fit indexes: root means square error of approximation (RMSEA) < 0.05, comparative fit index (CFI) < 0.90, Tucker-Lewis index (TLI) < 0.90, and standardized root mean squared residual (SRMR) < 0.08 (Hair et al., 2010).

We then tested the four expected mediation effects (H3 to H6). We estimated all expected indirect effects separately, by adding computed effects without affecting other regression coefficients or the general fit of the whole model. We combined the effect of the independent variable over the mediator and the effect of the mediator over the dependent variable. Such computed effects provided estimations of the independent indirect effects flowing through each mediator. They were obtained by multiplying the respective *a* and *b* paths under Baron and Kenny’s notation (Baron and Kenny, 1986).

Subsequently, we shifted from the explanatory to the predictive analysis, using the validated SEM model was as the initial structure to determine the best predictive model based on our data. We created a Dynamic Bayesian Network (DBN) through an extensive search for all possible SEM model extensions. Finally, we applied a Deep Reinforcement Learning approach to develop an automated fast-profiling method to evaluate respondents’ characteristics.

## Results

Supplementary Table S2 shows the standardized factor loadings for each item. The items generally showed a reasonable variation and were not overly skewed. Supplementary Table S3 contains the means and standard deviations of all measures. Regarding the difference between message frames (i.e., manipulation control), an analysis of variance showed that it was understood and recognized by participants (*p* = 0.001). Further analyses confirmed that group randomization was successful with respect to the baseline study variables and age (*p* > 0.39). Chi-square analysis revealed no significant differences in gender, education, and marital status across message groups (*p* > 0.68). In addition, message reading frequency was high (82.5% of participants read 14/15 messages), regardless of conditions (*p* = 0.21), and differently framed messages did not differ in whether they were systematically processed and perceived as involving and credible (*p* = 0.71). Finally, dropouts (*n* = 13) were not explained by conditions or baseline variables (*p* > 0.50).

### Structural equation model

**Figure 2:**
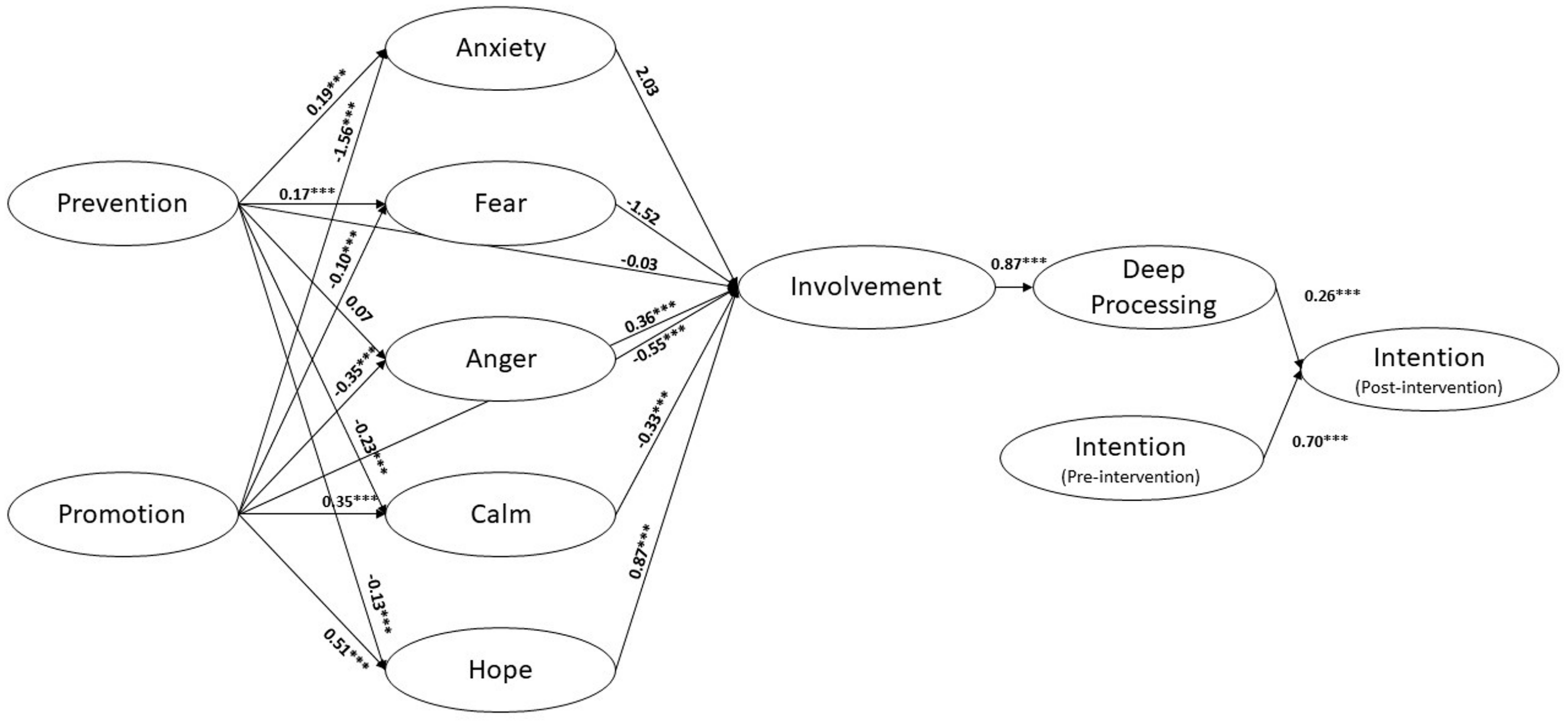
Theory-based structural equation model. *p<0.05, **p<0.01, ***p<0.001.

First, we checked the quality of the theory-based model using structural equation modeling and the goodness-of-fit index (Figure 2). The *χ*^2^ was significant, *χ*^2^(754) = 1936.50, *p* < 0.001. However, all other fit indices indicated that the model fitted the data well (RMSEA = 0.05, CFI = 0.93, TLI = 0.92). To rule out the possibility that competing models might explain our data better, we compared our theory-driven model with two other possible models. In one of them, perceived involvement *directly* affected post-intervention. This model was designed to rule out the possibility that higher involvement alone increased intention and thus test whether the Elaboration Likelihood Model’s central pathway was actually followed. In the second alternative model, we excluded the effect of intention before the intervention. In doing so, we wanted to test whether a simpler model, that omits a latent variable, is superior to our theory-driven model. The model comparison showed that both alternative models represented our data quite well, but worse than our theory-driven model (Supplementary Table S3).

### The mediation of emotions

We then tested our research hypotheses by looking more closely at the role of calm and hope triggered by the messages in promoting message involvement. As shown by the significant paths, calm decreased message involvement (*b* = −0.33), while hope increased it (*b* = 0.87), all *p* < 0.001. These results fully supported our H1 and H2_,_ respectively. Our model also showed that anger triggered by the messages was associated with lower message involvement, *b* = −0.55, *p* < 0.001. Evidently, messages that triggered higher levels of anger induced some reactance that was responsible for a reduced involvement with the messages. The decreased involvement due to calm, increased involvement due to hope, and reactance triggered by anger were consistent with the theories of emotional activation (Hovland et al., 1953; Leventhal, 1970), as well as with empirical findings emphasizing the role of positive emotions in promoting physical activity (Conner et al., 2015), and previous research combining emotion theories and message-based interventions related to health behavior (Carfora et al., 2021).

Both the prevention and promotion foci had an effect on calm and hope triggered by the messages (all *p* < 0.001). Specifically, promotion increased both calm (*b* = 0.35) and hope (*b* = 0.51), while prevention decreased both calm (*b* = −0.23) and hope (*b* = −0.13). To test our mediation hypotheses, we then estimated the total indirect effects of promotion and prevention on message involvement via calm and hope. As expected (H3), calm mediated the relationship between prevention focus and involvement. Participants with a higher prevention focus felt less calm after reading the messages, which was found to be functional as it led to higher message involvement (*b* = 0.07, *p* = 0.003). Again as expected (H4), participants with a higher promotion focus felt calmer after reading the messages, which proved detrimental as it led to lower message involvement (H2_B_), *b* = −0.11, *p* = 0.002.

Interestingly, and consistent with our hypotheses, hope mediated the relationship between regulatory focus and involvement with a pattern opposite to that of calm. Consistent with H5, participants with a higher prevention focus experienced less hope, leading to lower message involvement (H3_A_), *b* = −0.12, *p* < 0.001. Conversely, and consistent with H6, participants with a higher promotion focus experienced more hope, leading to higher message involvement (H3_B_), *b* = 0.44, *p* < 0.001.

In summary, the results of the mediation analyses fully supported our hypotheses and confirmed the presence of a multi-layered picture in which the two regulatory foci and the two positive emotions dynamically interacted with each other as they influenced perceived involvement in the messages.

### Dynamic Bayesian Network

**Figure 3:**
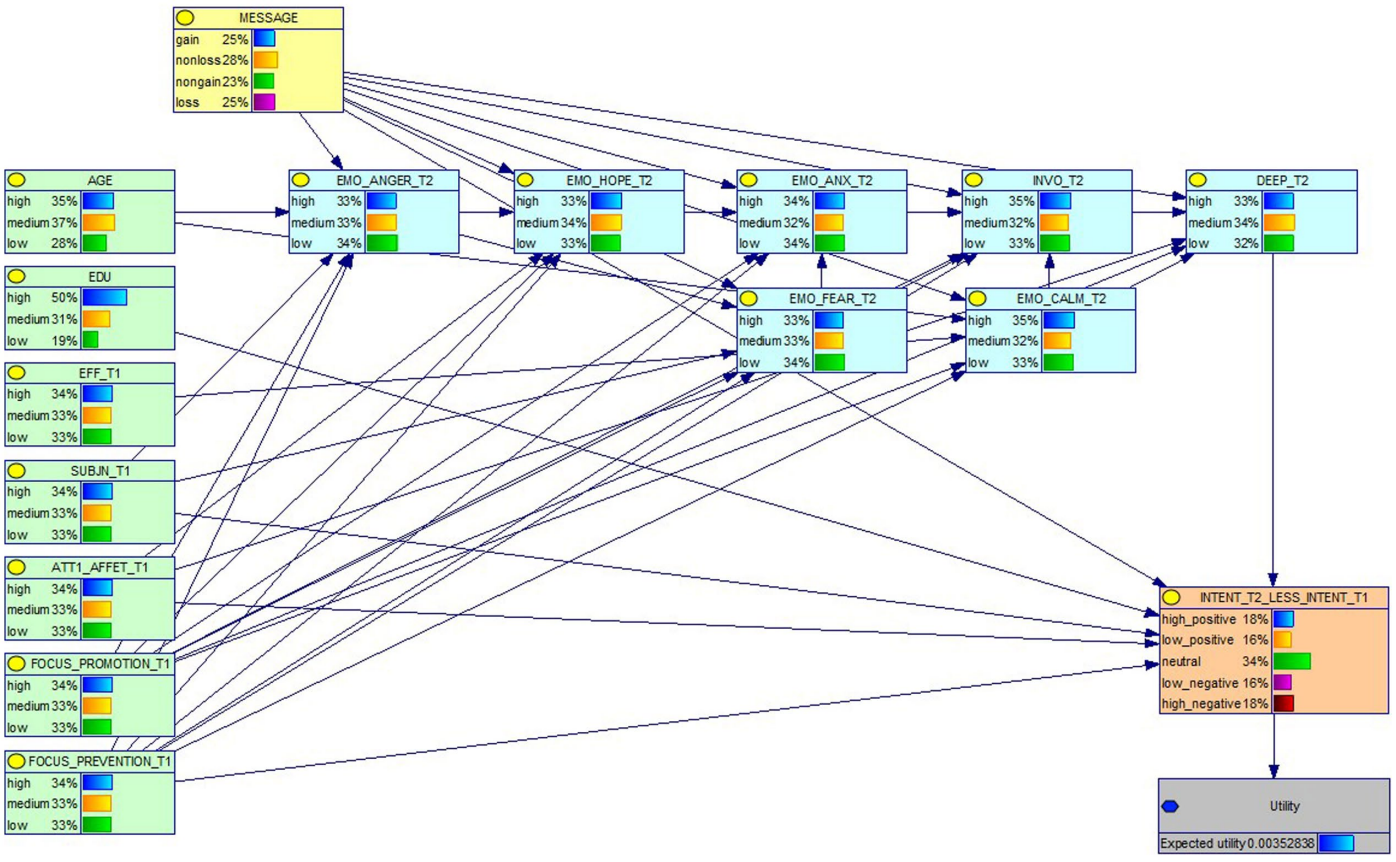
The elicited DBN structure.

We then moved from the explanatory to the predictive part of our analyses. In a first step, our theory-driven structural equation model was assumed to be the backbone structure of a Dynamic Bayesian Network (Dagum et al., 1995; Murphy, 2012). To make the network computationally manageable, we converted all variables into discrete variables, by calculating cut-offs that separated the three tertiles, i.e., coding the values into low, medium, and high. We then introduced additional socio-demographic variables (i.e., age, education, and gender) and the psychosocial variables of the Theory of Planned Behavior, i.e., attitude, perceived behavioral control, and social norm (Ajzen, 1991; Conner and Armitage, 1998). A selective search was then conducted in the space of possible structures, retaining only those variables that effectively improved the accuracy of the network, in terms of combined in-sample and out-of-sample predictive abilities. The structure of the Dynamic Bayesian Network was determined by an extensive automatic search as described in Catellani et al. (2022). Consistent with the intended use, candidate DBN structures were tested by using pre-intervention measures as evidence and then evaluating the conditional probability distribution for the target variable. The resulting predictor is illustrated in Figure 3.

Combined evaluations for in-sample and out-of-sample predictions of candidate structures were performed with actual data. Using the dataset of participants recruited through the Prolific platform and applying an out-of-sample (i.e., leave-one-out) testing method, the predictor yielded a multi-class Area-Under-Curve (AUC) (Ferri et al., 2009) value of 0.66. Applying the same testing method with a different dataset, collected with the cohort of 41 patients (see Participants and Procedure section), the multi-class AUC value of the predictor was 0.58. Such limited performance degradation was acceptable given the differences between the two populations, in particular in terms of age.

### Deep reinforcement learning and fast profiling policy

**Figure 4:**
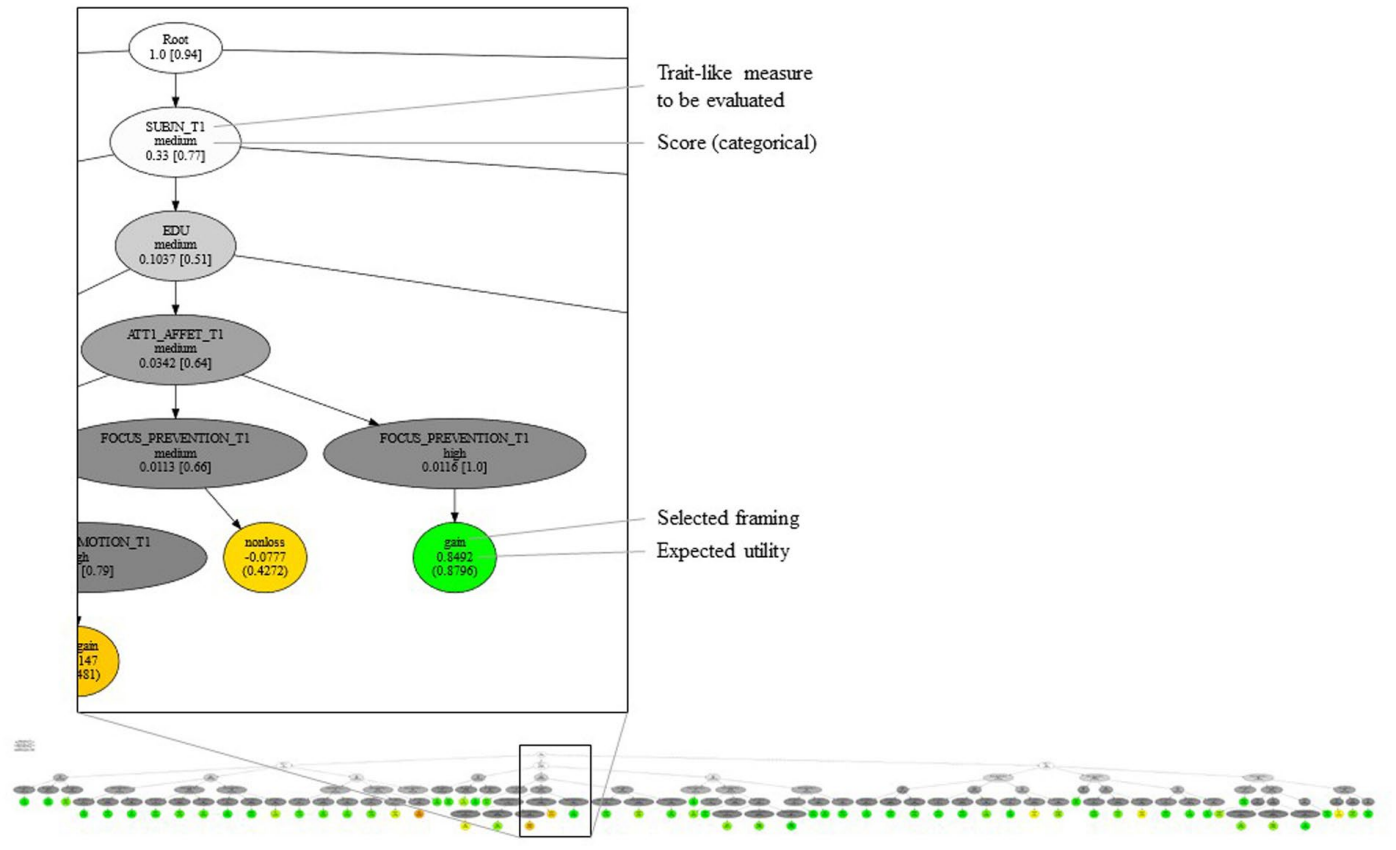
Fast profiling strategy as a tree-like structure (in the background), with one branch described in detail.

Our final goal was to make the results of this study applicable to real-world tools. Specifically, we aimed to develop an improved profiling policy that could help a chatbot identify the specific characteristics of a given recipient and consequently find the most appropriate message framing for that recipient. For this purpose, we relied on the AlphaZero technique (Silver et al., 2017) for Deep Reinforcement Learning (Li, 2017; François-Lavet et al., 2018). In general, AlphaZero performs a Monte Carlo Tree Search (Silver et al., 2016, 2017) under the guidance of a Deep Neural Network that is trained step-by-step to identify the moves with the best expected effectiveness depending on the state of the observations. In the variant of AlphaZero used in this study (Carfora et al., 2020), the Dynamic Bayesian Network was used to simulate the outcome of each move, namely, the receiver’s responses. At each simulated interaction step, trait-like measures were determined by sampling the probability distribution of the predictor, conditional on all trait-like measures observed up to that point. The result of the learning process was a Deep Neural Network-based representation of a probability distribution over the space of possible moves, conditional on the current state of the observed measures. A tree-like structure was obtained from the conditional distribution by selecting the move with the highest probability of effectiveness at each step and considering all possible outcomes from then on. Figure 4 shows a single branch of the tree-like structure (see Figure 1 in the Supplemental Materials for the full structure). The tree-like structure represents the fast profiling policy that was embedded in the chatbot of the RE-Hub-ILITY project, developed by Athics Srl. This chatbot is a smart conversational agent designed to promote physical activity. Thanks to the embedded fast-profiling strategy, the chatbot can profile the user within three to six interactions (instead of having to assess all the psychological constructs involved in our research). Once profiling is complete, the chatbot sends persuasive messages framed according to the best messaging policy identified in our study.

## Discussion

The present study provided several findings that increase our knowledge of how differently framed messages can influence people’s intentions to engage in physical activity. These findings can be grouped into three main areas, namely: (a) the soundness of our theory-driven model; (b) the impact of emotions in mediating the role of individual differences in perceived message involvement; (c) the fast profiling policy that can be achieved by applying Deep Reinforcement Learning to a predictive Dynamic Bayesian Network whose structure is rooted in a psychosocial theoretical framework. In what follows, we will comment separately on each group of results.

First, our theory-driven structural equation model has satisfactorily represented the observed data. The integration of the Elaboration Likelihood Model (Petty and Cacioppo, 1986; Petty and Briñol, 2011), the Self-Regulatory Model of Message Framing (Cesario et al., 2004), the Regulatory Focus Theory (Higgins, 1997), and theories of emotions (Hovland et al., 1953; Leventhal, 1970) proved to be a successful framework for investigating how persuasive messages can be used to promote physical activity. The model was robust and the relationships between variables were in line with our expectations. Individual differences in regulatory focus led to different emotions being experienced when reading messages, which in turn affected involvement, increasing or decreasing it in theoretically meaningful ways. In addition, greater involvement triggered deeper processing of the message, which in turn led to greater intention to engage in physical activity.

Regarding the role of emotions, our study yielded several significant results on their impact on message involvement, as well as their interaction with recipients’ regulatory focus. Overall, lower levels of calm and higher levels of hope increased message involvement. Regarding the decreasing role of calm, our results are consistent with previous research showing that failure to perceive minimal discomfort in relation to the current state of physical (in)activity is associated with ineffective promotion of physical activity (Hovland et al., 1953; Leventhal, 1970). At the same time, regarding the reinforcing role of hope our results are consistent with previous research showing that health communication triggers positive emotions in recipients (Conner et al., 2015).

Both calm and hope mediated the effect of regulatory focus on involvement with the messages and thus on deep processing and intentions. Participants with a higher prevention focus experienced lower levels of calm and this led to greater involvement. In contrast, participants with a higher promotion focus generally experienced higher levels of calm, which led to lower involvement, with in turn negatively impacted intention. Conversely, participants with a high prevention focus experienced such a positive emotion to a lesser extent and this resulted in lower involvement with the messages. Instead, participants with a high promotion focus experienced more hope, which directly translated into increased involvement.

These results suggest that a successful intervention with participants more focused on goal achievement (i.e., promotion) should consist of messages that at least partially reduce their experience of calm. Instead, a successful intervention with messages should aim to increase the level of hope experienced by participants with a stronger prevention focus. In this way, their tendency to feel easily hopeless could be counteracted, leading to increased involvement and deeper processing, which in turn would result in higher intention to engage in physical activity. To extend this line of research, future studies could further investigate the role of other psychological orientations of recipients. For example, examining the interaction of need for affect and need for cognition with message content and framing could lead to a more comprehensive understanding of the processes that influence people’s responses to persuasive messages (Aquino et al., 2020). This would contribute to the development of tailored communication strategies based on people’s preferences and cognitive styles.

The third important contribution of our study concerns the development of an innovative method for adapting persuasive communication to the characteristics of the recipients. All in all, the data analysis confirmed the soundness of our methodological approach. We developed a Causal Graphical Model (CGM) in the form of a Dynamic Bayesian Network (DBN) that incorporates our theory-based Structural Equation Model (SEM) and the message frame intervention as a structural backbone to obtain the best combination of in-sample explanatory power and out-of-sample predictive power.

The development of a Dynamic Bayesian Network based on a psychosocial theoretical framework provided a feasible way to use all available information to make persuasive communication more effective. Our predictor estimated the usefulness of each potential message frame and used this estimate to select the most promising frame according to the characteristics of the receiver. By improving the quality of the match between the message and the recipient, this data-driven technique enabled the extraction of useful prototypes that are consistent with established psychosocial theories and the identification of the message framing that is best suited to promote physical activity via a chatbot.

While SEM and mediation techniques allowed us to test adherence to psychological theories in a confirmatory way, Dynamic Bayesian Networks allowed us to take advantage of the increased predictive power of modern deep learning techniques. On the one hand, using the properties of predictive models has allowed us to include a larger number of variables in our analysis that play an important role in promoting physical activity. On the other hand, the use of an explanatory model as the backbone of the predictive model has given the model itself a strong theoretical orientation and helped to overcome the criticism often leveled at predictive models, namely that they “lack theory” (Shmuell, 2010; Hofman et al., 2021).

These findings suggest that social psychology and artificial intelligence can be integrated to develop effective online and personalized interventions to promote sustainable lifestyles that take into account individual preferences and orientations. Combining the explanatory power of theory-based structural equation models with the predictive power of data-driven artificial intelligence appears to be a promising strategy for effectively promoting sustainable lifestyles and supporting environmental policy through personalized and automated message-based interventions.

### Limitations and further developments

The biggest drawback of our study was that it relied on self-report measures. Such measures suffer from bias due to social desirability, they require participants to engage in a reasonable amount of introspection and they are prone to forgetfulness. For example, while participants completed the post-intervention questionnaire, they may have had difficulty recalling their immediate emotional reactions after receiving the persuasive messages. The use of wearable devices may, at least partially, overcome the present study’s reliance on self-report. Such devices provide robust measures of physical activity for individuals who are not risk of forgetfulness and social desirability.

A second limitation of our study could be the pre/post design. Although this design allowed us to attribute changes in intentions to our intervention, it lacked finer temporal resolution. Different emotional states may have been experienced during the two-week intervention, and tracking this could have yielded new and potentially more effective persuasion strategies. Future studies could consider measuring emotions experienced by recipients at different points during the intervention.

## Conclusion

In summary, in the present study we have combined the high explanatory power typical of theory-based models with the high predictive power of a data-driven approach. It is worth noting that the development of the predictor was based on the findings from the theory-based part. In this way, the explanatory power offered by theory-based approaches can be combined with the large predictive power of data-driven approaches. Taken together, the two parts of the present project can be seen as two sides of the same coin, providing a clear framework for implementing interventions to promote healthier lifestyles that are of great benefit to both individuals and society.

## Data availability statement

The raw data supporting the conclusions of this article will be made available by the authors, without undue reservation.

## Ethics statement

The studies involving human participants were reviewed and approved by the Commissione Etica per la Ricerca in Psicologia (CERPS), Catholic University of the Sacred Heart of Milan; Comitato Tecnico Scientifico, Istituti Clinici Scientifici Maugeri. The patients/participants provided their written informed consent to participate in this study.

## Author contributions

PC, MP, MB, and VC contributed to conception and design of the study. MB organized the database. MB and MP performed the statistical analysis. AN supervised the conception and design of the study. LB and MM contributed to the design of the study and the collection of the data. All authors wrote sections of the manuscript, contributed to manuscript revision, read, and approved the submitted version.

## Conflict of interest

The authors declare that the research was conducted in the absence of any commercial or financial relationships that could be construed as a potential conflict of interest.

## Publisher’s note

All claims expressed in this article are solely those of the authors and do not necessarily represent those of their affiliated organizations, or those of the publisher, the editors and the reviewers. Any product that may be evaluated in this article, or claim that may be made by its manufacturer, is not guaranteed or endorsed by the publisher.

## Footnotes

^1^https://www.prolific.com
