# Supplementary material for: A theory-based and data-driven approach to promoting physical activity through message-based interventions
Source: Front Psychol. 2023 Jul 27;14:1200304. doi: 10.3389/fpsyg.2023.1200304 (PMC10415075; doi:10.3389/fpsyg.2023.1200304)
Supplement: Supplementary file 1 [file Table_1.DOCX]

**A Theory-Based and Data-Driven Approach to Message Based-Interventions Promoting Physical Activity**

## Supplemental Materials

**Table 1.** *Standardized Factor Loadings of Study Measures.*

| Measures | Standardized  Factor Loading |
| --- | --- |
| Promotion Focus |  |
| I frequently imagine how I can achieve a state of “ideal health” | 0.60 |
| I think of good health as a key to a happy life | 0.57 |
| Doing healthy things gives me a sense of accomplishment | 0.77 |
| When I engage in healthy behaviour, I am pleased with myself | 0.73 |
| I would do anything to maintain a good, healthy body | 0.65 |
| I admire people who do things that make them very healthy | 0.64 |
|  |  |
| *Prevention Focus* |  |
| I often worry that I am not doing the best I can to improve my health | 0.75 |
| I often imagine myself being ill in the future | 0.57 |
| When I see people, who are very sick because they did not take care of their health, I get scared thinking that could be me in the future | 0.75 |
| I feel conscious about not doing all I ought to do to take care of my health | 0.62 |
| I often worry about not feeling as healthy as I used to be | 0.65 |
| Thinking about my health usually makes me worry | 0.70 |
|  |  |
| *Intention to Exercise Regularly at Time 1* |  |
| In the next month… | 0.97 |
| ... I intend to exercise regularly | 0.93 |
| ... I plan to exercise regularly | 0.94 |
| ... I want to exercise regularly |  |
|  |  |
| *Message Involvement* |  |
| The messages that I read... |  |
| ... got me involved | 0.86 |
| ... seemed relevant to me | 0.77 |
| ... were very interesting | 0.88 |
|  |  |
| *Deep Processing* |  |
| As I was reading the messages… |  |
| ... I've been thinking about what actions I might take myself based on what I've read | 0.81 |
| ... I found myself making connections between what I read in messages and information I heard/read in the past | 0.61 |
| ... I thought about how the information in the messages related to other things I know | 0.58 |
| ... I tried to think about the importance of the information offered by the messages for my daily life | 0.83 |
| ... I tried to relate the content of the information received with my behaviour | 0.78 |
|  |  |
| To what extent has reading the messages made you feel… |  |
| *Anger* |  |
| ... annoyed | 0.90 |
| ... irritated | 0.88 |
| ... bothered | 0.87 |
| *Anxiety* |  |
| ... agitated | 0.77 |
| ... worried | 0.74 |
| ... restless | 0.76 |
| *Fear* |  |
| ... afraid | 0.77 |
| ... scared | 0.82 |
| ... feared | 0.75 |
| *Hope* |  |
| ... hopeful | 0.82 |
| ... encouraged | 0.80 |
| ... optimistic | 0.85 |
| *Calm* |  |
| ... serene | 0.84 |
| ... calm | 0.78 |
| ... tranquil | 0.83 |
| Intention to Exercise Regularly at Time 2 |  |
| In the next month… |  |
| ... I intend to exercise regularly | 0.97 |
| ... I plan to exercise regularly | 0.93 |
| ... I want to exercise regularly | 0.94 |

**Table 2.** *List of Messages Used in the Message Intervention via the PsyMe App.*

| **Gain Messages** | **Non-Loss Messages** | **Non-Gain Messages** | **Loss Messages** |
| --- | --- | --- | --- |
| If you exercise regularly,  you will improve the function of your cardiovascular system. | If you exercise regularly, you will avoid  worsening the function of your cardiovascular system. | If you do not exercise regularly,  you will miss the chance to improve the function of your cardiovascular system. | If you do not exercise regularly,  you will worsen the function of your cardiovascular system. |
| If you exercise regularly, you will improve your fitness. | If you exercise regularly, will avoid worsening your fitness. | If you do not exercise regularly,  you will miss the opportunity to improve your fitness. | If you do not exercise regularly, you  will worsen your fitness. |
| If you exercise regularly,  you will enhance your muscle strength. | If you exercise regularly, you will avoid diminishing muscle strength. | If you do not exercise regularly,  you will miss the chance to improve your muscle strength. | If you do not exercise regularly,  you will diminish your muscle strength. |
| If you exercise regularly, you will feel more agile. | If you exercise regularly, you will avoid feeling less agile. | If you do not exercise regularly,  you will miss the chance to feel more agile. | If you do not exercise regularly,  you will feel less agile. |
| If you exercise regularly, you could improve your sex life. | If you exercise regularly, you could avoid making your sex life worse. | If you do not exercise regularly,  you may miss the chance to improve your sex life. | If you do not exercise regularly,  you could make your sex life worse. |
| If you exercise regularly,  you will increase your good mood. | If you exercise regularly, you will avoid reducing your good mood. | If you do not exercise regularly,  you will lose the chance to increase your good mood. | If you do not exercise regularly,  you will reduce your good mood. |
| If you exercise regularly, you will increase your emotional well-being. | If you exercise regularly, you will avoid reducing your emotional well-being. | If you do not exercise regularly,  you will miss the opportunity to increase your emotional well-being. | If you do not exercise regularly,  you will reduce your emotional well-being. |
| If you exercise regularly, you will increase your vitality. | If you exercise regularly, you will avoid diminishing your vitality. | If you do not exercise regularly,  you will miss the opportunity to increase your vitality. | If you do not exercise regularly,  you will decrease your vitality. |
| If you exercise regularly, you will feel more in tune with your body. | If you exercise regularly, you will avoid feeling less in tune with your body. | If you do not exercise regularly, you will lose the opportunity to feel more in tune with your body. | If you do not exercise regularly,  you will feel less in tune with your body. |
| If you exercise regularly, you will feel more satisfied. | If you exercise regularly, you will avoid feeling more dissatisfied. | If you do not exercise regularly,  you will miss the chance to feel more satisfied. | If you do not exercise regularly,  you will feel more dissatisfied. |
| If you exercise regularly, you will increase your well-being when you are with other people. | If you exercise regularly, you will avoid diminishing your well-being when you are with other people. | If you do not exercise regularly,  you will lose the ability to increase your well-being when you are with other people. | If you do not exercise regularly,  you will decrease your well-being when you are with other people. |
| If you exercise regularly, you will be more appreciated by others. | If you exercise regularly, you will avoid being less appreciated by others. | If you do not exercise regularly,  you will miss the chance to be more appreciated by others. | If you do not exercise regularly,  you will be less appreciated by others. |
| If you exercise regularly, you will feel more comfortable with other people. | If you exercise regularly, you will avoid feeling less comfortable with other people. | If you do not exercise regularly,  you will lose the opportunity to feel more comfortable with other people. | If you do not exercise regularly,  you will feel less comfortable with other people. |
| If you exercise regularly, you will feel more approved by other people. | If you exercise regularly, you will avoid feeling less approved by other people. | If you do not exercise regularly,  you will miss the chance to feel more approved by other people. | If you do not exercise regularly,  you will feel less approved by other people. |
| If you exercise regularly, you will increase your independence from others. | If you exercise regularly, you will avoid increasing your dependence on others. | If you do not exercise regularly,  you will miss the chance to increase your independence from others. | If you do not exercise regularly,  you will increase your dependence on others. |

**Table 3.** *Descriptive Statistics of Latent Variables.*

|  | Gain | | Non-Loss | | Non-Gain | | Loss | | Entire Sample | |
| --- | --- | --- | --- | --- | --- | --- | --- | --- | --- | --- |
| Variable | M | SD | M | SD | M | SD | M | SD | M | SD |
| **Time 1** |  |  |  |  |  |  |  |  |  |  |
| Prevention | 5.04 | 1.22 | 4.94 | 1.00 | 5.00 | 0.90 | 5.09 | 0.92 | 5.32 | 0.90 |
| Promotion | 4.27 | 1.24 | 4.40 | 1.33 | 4.27 | 1.20 | 4.42 | 1.16 | 4.17 | 1.23 |
| Intention | 4.59 | 1.24 | 4.30 | 1.65 | 4.64 | 1.59 | 4.19 | 1.66 | 5.52 | 1.52 |
| **Time 2** |  |  |  |  |  |  |  |  |  |  |
| Involvement | 4.81 | 1.18 | 4.74 | 1.18 | 4.54 | 1.34 | 4.38 | 1.43 | 4.82 | 1.24 |
| Deep Processing | 4.96 | 1.13 | 4.93 | 1.17 | 4.63 | 1.13 | 4.76 | 1.21 | 5.08 | 1.08 |
| Anger | 1.44 | 0.61 | 1.77 | 0.86 | 1.90 | 1.06 | 2.01 | 0.88 | 1.71 | 0.89 |
| Anxiety | 1.53 | 0.61 | 1.69 | 0.58 | 1.80 | 0.71 | 2.09 | 0.85 | 1.69 | 0.72 |
| Fear | 1.30 | 0.62 | 1.43 | 0.57 | 1.61 | 0.65 | 1.84 | 0.79 | 1.50 | 0.66 |
| Calm | 3.02 | 0.90 | 2.89 | 0.84 | 2.83 | 0.85 | 2.46 | 0.98 | 2.96 | 0.86 |
| Hope | 2.92 | 0.98 | 2.50 | 0.95 | 2.50 | 0.79 | 2.28 | 0.82 | 2.78 | 0.92 |
| Intention | 5.16 | 1.41 | 4.39 | 1.53 | 4.83 | 1.67 | 4.28 | 1.78 | 5.54 | 1.45 |

**Table 4.** *Results of Model Comparisons among our Theory-Based Model and the Two Alternative Models.*

| Model | RMSEA | CFI | TLI | Df | AIC | BIC | χ^2^ | Δχ^2^ | Δdf | p |
| --- | --- | --- | --- | --- | --- | --- | --- | --- | --- | --- |
| Theory-Based | .05 | .93 | .92 | 754 | 59258 | 59899 | 1936.5 | - | - | - |
| Direct Involvement | .05 | .93 | .92 | 753 | 59260 | 59905 | 1936.0 | 0.54 | 1 | .461 |
| Without Intention T1 | .06 | .90 | .90 | 755 | 59691 | 60327 | 2371.1 | 434.65 | 1 | <.001 |

**Figure 1**. Fast Profiling Strategy as a Tree-Like Structure.
